# Research on the Heating Process of CFRP Circular Tubes Based on Electromagnetic Induction Heating Method

**DOI:** 10.3390/polym15143039

**Published:** 2023-07-14

**Authors:** Jiazhong Xu, Yunfei Gu, Tianyu Fu, Xiaobing Zhang, Hao Zhang

**Affiliations:** 1 Key Laboratory of Advanced Manufacturing and Intelligent Technology Ministry of Education, School of Automation, Harbin University of Science and Technology, Harbin 150080, China; 2 Key Laboratory of Advanced Manufacturing and Intelligent Technology Ministry of Education, School of Mechanical and Power Engineering, Harbin University of Science and Technology, Harbin 150080, China; guyunfei2023@163.com; 3 Department of Intelligent Equipment, Changzhou College of Information Technology, Changzhou 213164, China; 18704613530@163.com; 4 HaiKong Composite Materials Technology Co., Ltd., Jinan 250000, China; zxbhk@126.com (X.Z.); 280288510@163.com (H.Z.)

**Keywords:** electromagnetic induction heating, mold, temperature field, CFRP

## Abstract

Based on the electromagnetic induction heating method, heating and curing of Carbon Fiber Reinforced Polymer (CFRP) have the advantages of high energy utilization and no pollution. However, in the heating process, both the material weaving structure and mold material can affect the temperature field. Therefore, in this study, an electromagnetic heating finite element analysis model for CFRP circular tubes was established based on the equivalent electromagnetic thermal characteristics of CFRP. The study investigated the temperature rise mechanism of the material weaving structure under the magnetic field, and explored in-depth the influence of molds made of 45# steel and glass fiber-reinforced plastic (FRP) on the heating process of CFRP. The CFRP circular tubes with weaving structures of 89-degree winding angle, 45-degree winding angle, and plain weave were studied. The study found that when the metal mold was heated, the CFRP structure had almost no effect on the temperature distribution. However, when the glass fiber-reinforced plastic mold was heated, the temperature field changed with the CFRP structure, and the more fiber cross points, the more uniform the temperature field. The accuracy of the finite element model was verified through experiments. The aim of this research is to provide theoretical guidance for actual industrial production.

## 1. Introduction

Carbon fiber reinforced polymer (CFRP) combines the advantages of lightweight, high strength, corrosion resistance, and strong plasticity, making it a powerful promoter for sustainable development [1,2,3,4,5]. In the past decades, it has been widely used in fields such as aerospace, medical equipment, and automobile manufacturing. With the increasing awareness of environmental protection and efficient manufacturing, the demand for CFRP is also constantly rising [6,7,8]. Correct and uniform temperature field distribution is one of the keys to improving the molding quality of CFRP. For CFRP circular tube structures, the common heating devices are autoclave heating high-temperature curing chamber and steam internal curing, where heat is conducted to the overall composite material through its surface, resulting in uneven temperature field distribution in the thickness direction and waste of energy due to a large amount of heat dissipated into the air during the heating process [9,10,11]. Electromagnetic induction heating, as a heating method that is pollution-free, occupies a small equipment footprint, is easy to operate, and has no contact with the heated workpiece, has been widely used in fields such as metal smelting and welding [12,13,14,15,16]. In order to achieve in-situ curing of CFRP, and due to the conductivity of carbon fibers, the combination of electromagnetic induction technology with CFRP curing has become a viable alternative to traditional CFRP curing heating methods. In the process of CFRP electromagnetic induction heating and curing, the material’s winding structure and mold material will affect the temperature field distribution of the induced heating composite material, which in turn affects the molding effect of the composite material.

There have been significant advances in the research on the induction heating of CFRP both domestically and internationally, and many scholars have conducted in-depth studies on the heat generation mechanism, temperature field distribution, and influencing factors during the induction heating of CFRP. Wasselynck et al. [17] proposed a new multiscale homogenization method to calculate the electric field and current distribution of carbon fiber composite materials under alternating magnetic fields. The transverse conductivity and electric and magnetic equivalent properties of carbon fiber composite materials were calculated using finite element methods at the microscale. At the macroscale, an equivalent induction heating impedance network model was established to study the anisotropy of carbon fiber composite materials in induction heating. Lundström et al. [18] established an axisymmetric model for diagonal braided CFRP plates to analyze the dependence of material thermal conductivity, specific heat, and electrical conductivity on temperature and their influence on induction heating. The study found that the temperature dependence of material properties has a significant impact on the heating rate, where the thermal conductivity and specific heat are basically proportional to temperature and have a large temperature dependence, while the electrical conductivity has a small dependence. Fu et al. [19] provided a microscale model of plain weave CFRP plates for electromagnetic induction heating and analyzed the distribution of eddy current and temperature fields at the microscale during the heating process. Bruce et al. [20] input an ascending temperature model that can predict the transient heat distribution inside different carbon fiber composite structures into finite element thermal conduction analysis, which realizes a finite element model and analysis method to predict the internal transient heat distribution of layered plates on different molds. The above scholars have studied the temperature field variation laws of braided structure CFRP plates and the influence of different material parameters on the temperature field, but there is no research on CFRP induction heating on different rotating molds.

Due to the influence of the weave structure and mold material on the temperature field distribution during the induction heating process of CFRP circular tubes, this paper investigates the induction heating mechanism of CFRP circular tubes and the effects of different mold materials and weave structures on their heating performance. Firstly, a finite element analysis model of CFRP circular tubes rotating under induction heating is established based on the equivalent electromagnetic thermal properties of CFRP. Secondly, taking the plain weave structure of CFRP circular tubes as an example, the effects of no mold and different mold materials on the heating performance of CFRP circular tubes are simulated and calculated. Thirdly, the heating process of CFRP circular tubes with different structures is simulated and analyzed, including the effects of the winding structure on the heating mechanism and the coupled effects of different mold materials on the temperature field variation. Finally, the CFRP circular tube induction heating experimental platform verifies that the model established in this paper can realize the study of material heating mechanism and temperature field distribution, and provide theoretical guidance for actual industrial production.

## 2. Materials and Methods

### 2.1. The Establishment of Theoretical Model

#### 2.1.1. Maxwell’s Equations

When an alternating current is passed through a coil, an alternating magnetic field is generated at the center and around the coil, as shown in Equation (1), where ∇ is the Hamiltonian operator, *H* is the magnetic field strength (A/m), and *J* is the current density (A/m^2^) passing through the coil.

(1) ∇×H=J

When the CFRP tube and mold rotate, the cutting of alternating magnetic lines of force can generate an electric potential. Since the potential cannot be directly calculated, the magnetic vector potential *A* (Wb/m) needs to be introduced, as shown in Equation (2). In the frequency domain, the electric potential generated by the mold and CFRP tube can be expressed as shown in Equation (3), where *B* is the magnetic flux density (T) and *E* is the electric potential (V/m).

(2) B=∇×A

(3) E=−jωA

To generate a current, a closed circuit must be formed in the mold and CFRP tube. The current density *J_t_* (A/m^2^) is shown in Equation (4), where σ is the conductivity tensor, *D* is the electric displacement vector (C/m^2^), *v* is the velocity (m/s), σE is the electromagnetic induction contribution, which jωD is almost negligible compared to the source current, and σv×B is the Lorentz contribution.

(4) $$J_{t}=\sigma E+j\omega D+\sigma v\times B$$

The main way of generating heat in the mold and CFRP circular tube is Joule heating, which can be described by the following Equation (5):

(5) $$Q=\frac{J_{t}^{2}}{\sigma }$$

#### 2.1.2. Heat Transfer Equations

After heat is generated, it transfers within the material or between different materials. The governing equation for this process is shown in Equation (6), where *ρ* is the density of the material (kg/m³), *C_p_* is the specific heat capacity of the material (J/(kg·K)), λ is the thermal conductivity tensor of the material, ∇T is the temperature difference (°C), μ is the resin flow velocity (m/s), where $\rho C_{p}\frac{\partial T}{\partial t}$ is the temperature as a function of time, $\nabla \cdot \left(-\lambda \nabla T\right)$ is the contribution of thermal conduction, and $\rho C_{p}\mu \cdot \nabla T$ is the contribution of convective heat transfer.

(6) $$\rho C_{p}\frac{\partial T}{\partial t}+\nabla \cdot \left(-\lambda \nabla T\right)+\rho C_{p}\mu \cdot \nabla T=Q$$

#### 2.1.3. Boundary Conditions

In the induction heating model presented in this paper, it is necessary to consider the influence of heat flux and thermal radiation on heat transfer in materials. The governing equations for this process are shown in Equations (7) and (8), where α is the heat transfer coefficient (W/m^2^·K) determined by the boundary type and surface properties, *T* is the temperature of the external air (°C), *T*_0_ is the surface temperature of the heated material (°C), γ is the Stefan-Boltzmann constant, and ε is the emissivity of the material surface.

(7) $$-\lambda \nabla T=\alpha \left(T-T_{0}\right)$$

(8) $$-\lambda \nabla T=\gamma \varepsilon \left(T^{4}-T_{0}^{4}\right)$$

### 2.2. Establishment of Finite Element Model

#### 2.2.1. Establishment of Geometric Model

**Figure 1:**
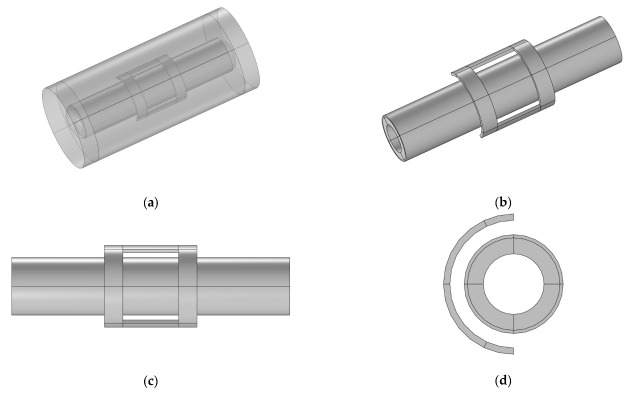
Geometric model of induction heating; (**a**) overall geometric model, (**b**) geometric model with air domain removed, (**c**) The front view of the geometric model with the air domain removed, (**d**) The side view of the geometric model with the air domain removed.

This paper employs the COMSOL Multiphysics software to establish a layered plate model of CFRP tubes, which is a common approach for modeling composite materials. COMSOL Multiphysics is a large-scale advanced numerical simulation software suitable for electromagnetic induction heating simulations involving rotational motion. In the study, the mold and CFRP tubes are in a rotating state, with the tubes including a winding plain weave structure and fiber winding structures (winding angles of 89 degrees and 45 degrees). The tubes and mold act as the rotor, while the coil serves as the stator. Since the finite element method does not support rotation, a combination of the moving mesh method and the rotating machinery magnetic physics interface is employed to simulate the rotational motion of the tubes. The moving mesh method is a commonly used numerical simulation technique in the computational domain, which adjusts the mesh in the calculation domain to adapt to the evolution and deformation of the problem. The Rotating machinery magnetic physics interface is a physical interface used in COMSOL to simulate the interaction between magnetic fields and mechanical motion in rotating machinery systems. Figure 1 shows the geometric model of the induced heating with a mold.

**Figure 2:**
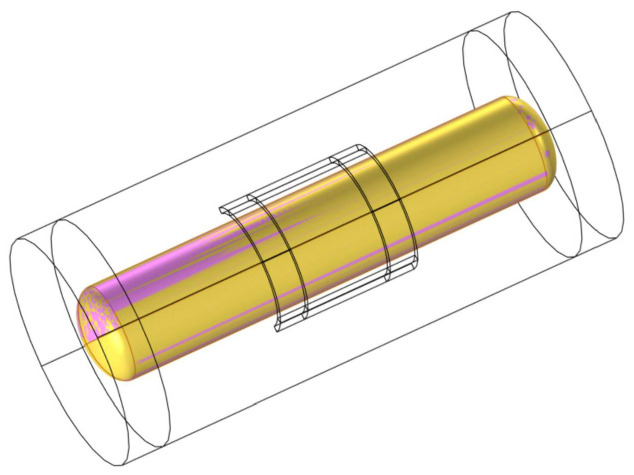
Schematic diagram of consistent boundary pairs.

**Figure 3:**
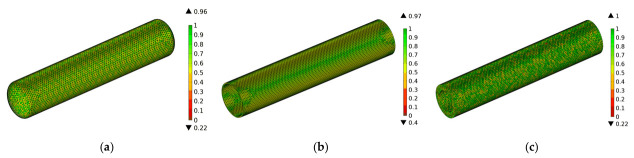
Mesh quality diagram; (**a**) Mesh quality diagram of consistent boundary pairs (**b**) Mesh quality diagram of CFRP tube (**c**) Mesh quality diagram of the mold.

**Figure 4:**
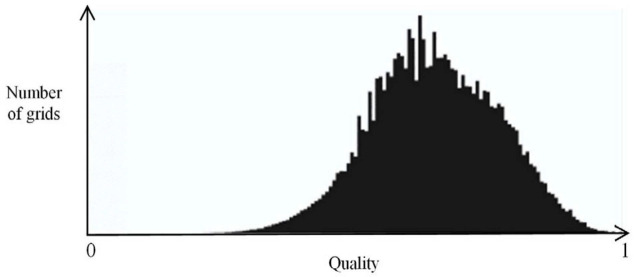
Histogram of mesh quality.

To ensure the continuity of the magnetic field during the rotation process, it is necessary to form consistent boundary pairs at the interface between the rotating and stationary domains. This allows the magnetic field generated by the coil to act on the rotating domain. Figure 2 illustrates a schematic diagram of the consistent boundary pairs, where the yellow and pink mixed boundaries represent the consistent boundary pairs. The consistent boundary pairs are composed of surface elements, and the mesh is divided using a free triangular mesh for the boundary region while a free tetrahedral mesh is used for the remaining parts. The quality of the mesh directly affects the accuracy and efficiency of the calculations, and a good mesh quality ensures the accuracy of the results. The closer the numerical value of the mesh quality is to 1, the better the mesh quality. Figure 3 shows the mesh quality, where (a) represents the mesh quality of the consistent boundary pairs, (b) represents the mesh quality of the CFRP circular tube, and (c) represents the mesh quality of the mold. It can be observed from the figure that there are relatively few meshes with a quality below 0.5. Figure 4 shows a histogram of the geometric mesh quality, which intuitively indicates that the mesh quality is mainly concentrated between 0.5 and 0.8, indicating a good mesh quality for the mesh division.

#### 2.2.2. Setting of Physical Parameters

In the geometric model, the CFRP circular tube has dimensions of φ29 × 15 mm with a thickness of 1 mm, while the mold has dimensions of φ19 × 15 mm with a thickness of 5 mm. The coil is an arc-shaped coil with an angle of 180 degrees. When there is no mold, the number of turns is 100, the current is 20 A, and the frequency is 25 KHz. When using a mold made of glass fiber-reinforced plastic (FRP), the parameters are set to be the same as those without a mold. When using a mold made of 45# steel, the number of turns is 50, the current is 10 A, and the frequency is 13 KHz. The distance between the coil and the CFRP circular tube is 3.5 mm. The ambient temperature is 20 °C, and the rotation speed is 5 rpm. Since the simulated induction heating environment is indoors with no strong convective air, the value can be set to 5. The radiation rates of the CFRP composite material and the mold are different and set to 0.3 and 0.2, respectively. To characterize the different structural features of the CFRP circular tube in the laminate model, such as the plain weave and fiber winding structures (winding angles of 89 degrees and 45 degrees), Converting the Cartesian coordinate system to cylindrical coordinates allows us to characterize the conductivity and thermal conductivity of CFRP tubes with different structural features. The anisotropic parameters are represented in diagonal form, where the first term corresponds to the rr component, the second term corresponds to the phiphi component, and the third term corresponds to the zz component. The material parameters for CFRP, coils, and air are obtained from Gu et al. [21], while the material parameters for 45# steel mold come from Park et al. [22], and the material parameters for fiberglass reinforced plastic (FRP) mold are obtained from Xu et al. [23] The specific parameters are shown in Table 1.

## 3. Results

### 3.1. Induction Heating Mechanism for Rotating CFRP Circular Tubes

**Figure 5:**
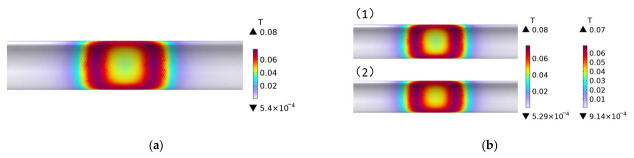
Magnetic flux density distribution with and without molds; (**a**) the magnetic flux density distribution of plain weave CFRP circular tubes wound without molds, (**b**) the magnetic flux density distribution of plain weave CFRP circular tubes wound with FRP molds.

Figure 5a,b illustrates the magnetic flux density distribution of plain-weave CFRP circular tubes wound without molds and with FRP molds, respectively, during the induction heating process. In the figures, (1) represents the CFRP circular tube example, corresponding to the color bar on the left, and (2) represents the mold example, corresponding to the color bar on the right. The arrangement of the CFRP circular tube and the mold in the figures is the same. As can be seen from the figures, the magnetic flux distribution of the CFRP circular tube on both the mold and without the mold appears rectangular, consistent with the projection of the coil on the tube, and the numerical values are also very similar. Figure 5b shows that the expression of the CFRP circular tube with the FRP mold is almost the same numerically, which is due to the fact that the magnetic conductivity of the CFRP circular tube is the same as that of the FRP mold.

**Figure 6:**
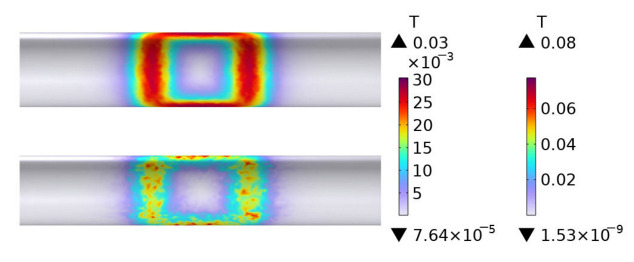
Magnetic flux density distribution of 45# steel and plain weave CFRP circular tube.

Figure 6 shows the magnetic flux density distribution pattern of the woven plain weave structure on the 45# steel mold during the rotating induction heating process. From the figure, it can be seen that both the magnetic flux distribution of the 45# steel mold and the CFRP circular tube present a rectangular shape, which is consistent with the projection of the coil on the circular tube. In terms of numerical values, the magnetic flux density of the 45# steel mold is significantly higher than that of the CFRP circular tube, which is due to the higher relative magnetic permeability of the metal mold and its stronger magnetic conductivity.

**Figure 7:**
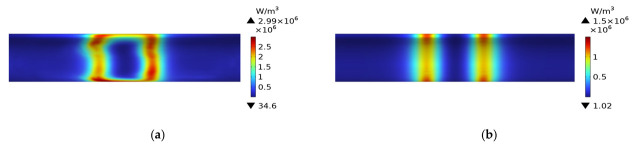

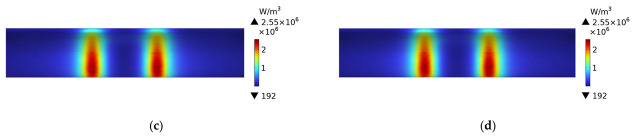
Distribution of eddy current losses of plain weave CFRP cylindrical tubes wound without molds during the process of rotating induction heating; (**a**) 1 s, (**b**) 60 s, (**c**) 600 s, (**d**) steady state.

Figure 7 shows the distribution of eddy current losses of plain weave CFRP circular tubes wound without molds during the process of rotating induction heating, from initial heating to steady-state. The figure indicates that at 1 s, the eddy current losses of the CFRP circular tube appear in a rectangular shape, consistent with the projection of the coil onto the circular tube. At 60 s, it can be observed that the heat generation is concentrated at the position directly facing the coil. From 60 s onwards, until steady-state, the distribution of losses remains constant and consists of mutually parallel vertical lines. It is evident from the numerical values that the heat generation remains constant after 600 s.

**Figure 8:**
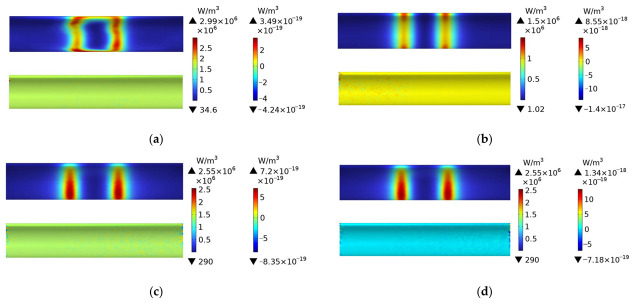
Distribution of eddy current losses of plain weave CFRP cylindrical tubes wound with FRP molds during the process of rotating induction heating; (**a**) 1 s, (**b**) 60 s, (**c**) 600 s, (**d**) steady state.

Figure 8 shows the distribution of eddy current losses of plain weave CFRP cylindrical tubes wound with FRP molds during the process of rotating induction heating, from initial heating to steady-state. From the figure, it can be observed that the FRP mold does not generate heat. When compared to Figure 7, it can be noticed that the distribution and numerical values of eddy current losses of the CFRP cylindrical tube in Figure 8 are almost identical to those in Figure 7. This is because only closed loops of current can generate heat, and FRP is non-conductive. Therefore, during the heating of the CFRP cylindrical tube with an FRP mold, the heat source originates entirely from the CFRP itself.

**Figure 9:**
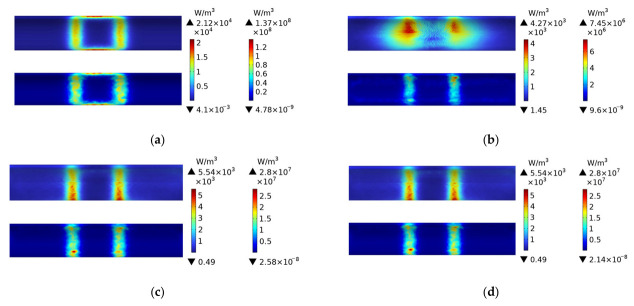
Eddy current loss distribution of 45# steel mold and plain weave CFRP circular tube; (**a**) 1 s, (**b**) 60 s, (**c**) 600 s, (**d**) steady state.

Figure 9 shows the distribution of eddy current losses in a plain weave structure CFRP circular tube wound around a 45# steel mold during the process of induction heating from initial heating to steady state. The figure indicates that at 1 s, the eddy current losses of the CFRP circular tube are distributed in a rectangular pattern, with the projection of the losses located directly in front of the coil. From 60 s to steady-state, the distribution of losses gradually changes to parallel vertical lines that remain constant. Numerically, it is apparent that the heating body, the 45# steel mold, is four orders of magnitude higher in comparison to the CFRP circular tube. This fundamental difference is due to the much higher magnetic permeability of metal than that of CFRP, which results in the coil’s power mainly acting on the metal.

Comparing Figure 9 to Figure 8, it is observed that the distribution of losses in the CFRP circular tube is almost identical in both figures, except for a slight difference at 60 s. According to Table 1, the anisotropic parameters of the plain weave structure are mainly distributed along the axis and circumference of the circular tube. In Figure 8, the CFRP circular tube is the heating body and dominates the heat generation, while the FRP mold does not generate heat, resulting in a greater amount of heat at the central position. In contrast, in Figure 9, the 45# steel mold is the heating body, with isotropic material properties. The heat generated is concentrated at two vertical line positions, while the CFRP circular, as a secondary heating body, leads to a small amount of heat in the middle position.

**Figure 10:**
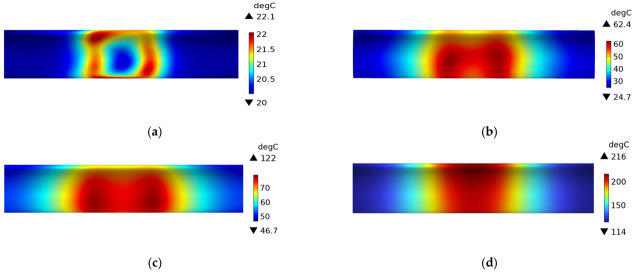
Temperature field distribution of a plain weave CFRP circular tube without a mold; (**a**) 1 s, (**b**) 60 s, (**c**) 600 s, (**d**) steady state.

Figure 10 shows the temperature field distribution of a plain weave CFRP circular tube without a mold during the induction heating process from initial heating to steady state. At 1 s, the temperature field exhibits an irregular rectangular shape and is located at the position directly facing the coil. As time progresses, the area gradually expands and transforms into an elongated ellipse, ultimately forming a rectangular shape in steady state. It can be observed that at 60 s, the position with the highest temperature shifts from the position directly facing the coil to the position directly facing the two vertical sides of the coil. Corresponding to Figure 7, at 1 s, the heat generation is in the form of a rectangle, and at this point, temperature conduction has just begun. The temperature rise starts from the position facing the coil, resulting in a rectangular temperature distribution. At 60 s, the location of heat generation shifts to the position facing the two vertical sides of the coil. The temperature field further expands into an elongated ellipse, with the highest temperature values distributed at the positions facing the two vertical sides. At 600 s, the heat source becomes two parallel vertical lines, and the temperature field distribution shows that the temperature is highest at the position of the two lines, gradually decreasing in other regions. In the steady state, the temperature of all regions of the circular tube has risen, returning to the initial state, with the temperature at the center of the coil being the highest and gradually decreasing along the axis on both sides.

**Figure 11:**
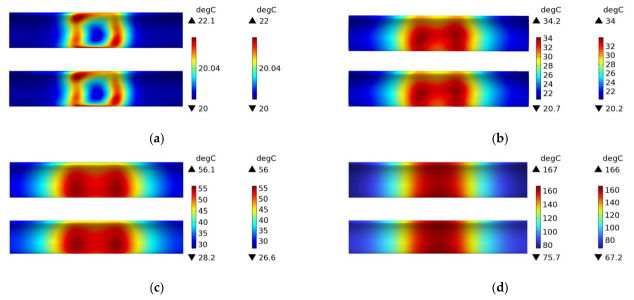
Temperature field distribution of a plain weave CFRP circular tube with a FRP mold; (**a**) 1 s, (**b**) 60 s, (**c**) 600 s, (**d**) steady state.

Figure 11 shows the temperature field distribution of a plain weave CFRP circular tube with an FRP mold during the induction heating process from initial heating to steady state. Compared with Figure 10, it can be observed that the temperature field distribution of the CFRP circular tube is almost identical, except that the temperature values in Figure 11 are lower than those in Figure 10. This is because the same heating source was used in both Figure 10 and Figure 11, but in Figure 10, there was no mold to share the heat, so the heat was only transferred within the CFRP circular tube. In contrast, in Figure 11, the mold shared some of the heat, resulting in the observed difference in temperature values.

**Figure 12:**
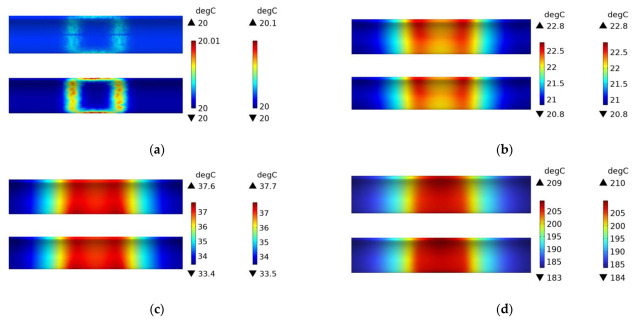
Temperature field distribution of 45 # steel mold and plain weave CFRP circular tube; (**a**) 1 s, (**b**) 60 s, (**c**) 600 s, (**d**) steady state.

Figure 12 shows the temperature field distribution of the 45# steel mold wrapped with plain weave CFRP tube during the induction heating process from initial heating to steady state. It can be observed that the temperature field distribution gradually transitions from a rectangular shape to higher temperatures at the two ends and lower temperatures in the middle, and eventually reaches a steady state where the middle temperature is higher and the two ends are lower, with an overall regular rectangular distribution. The reason for this process can be inferred from the heat distribution shown in Figure 9: the heat source is the source of all temperature, and at 1 s, due to the steel mold being the primary heat source, its temperature field distribution is more significant and with higher numerical values than the CFRP tube. At 60 s, the heat distribution is higher at the two ends and lower in the middle, with the temperature mainly conducted to the two ends of the tube while the middle temperature remains relatively low. At 600 s, the temperature on both sides of the tube gradually rises, and the middle temperature begins to increase gradually, reaching the highest point at steady state. The higher temperature of the 45# steel mold compared to the tube at steady state is due to the fact that temperature conduction takes time and the thermal conductivity coefficients between the two different materials are different, resulting in different conduction speeds. Compared with Figure 11, it can be seen that the temperature field distribution of the metal mold is more uniform than that of the FRP mold and CFRP tube.

### 3.2. Experimental Verification and Analysis

**Figure 13:**
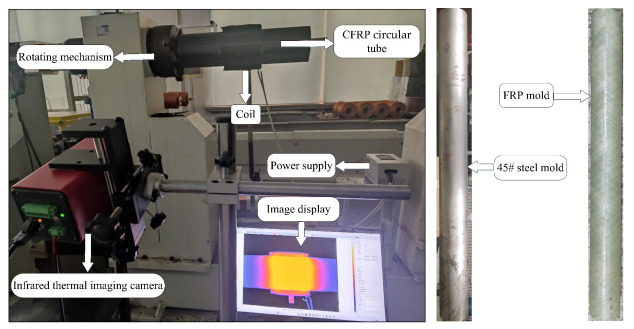
Experimental device diagram of CFRP circular tube.

To validate the accuracy of the finite element model established in this study, experimental tests were conducted to investigate the temperature distribution during electromagnetic induction heating of the CFRP circular tube. The experimental setup for induction heating of the CFRP circular tube is shown in Figure 13. The rotational mechanism provides power to rotate the CFRP circular tube against the mold, and the FOTRIC 618C infrared camera is used to record the heating process. The coil is powered by a control power supply. The dimensions and parameters of the coil structure in the experiment are consistent with those set in the simulation.

**Figure 14:**
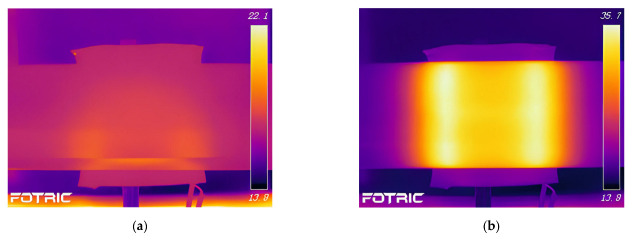

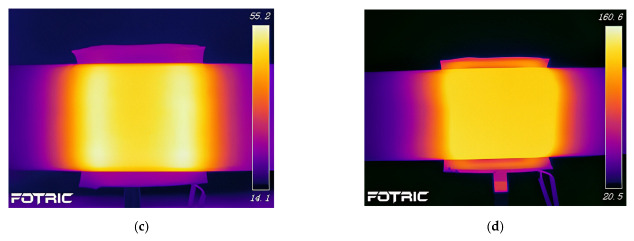
Experimental temperature field distribution of FRP mold and plain weave CFRP circular tube; (**a**) 1 s, (**b**) 60 s, (**c**) 600 s, (**d**) steady state.

**Figure 15:**
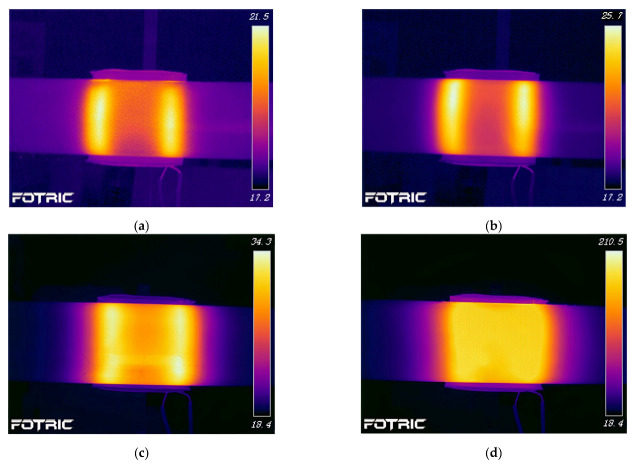
Experimental temperature field distribution of 45# steel mold and plain weave CFRP circular tube; (**a**) 1 s, (**b**) 60 s, (**c**) 600 s, (**d**) steady state.

Figure 14 shows the variation of temperature distribution in the fiberglass mold and CFRP circular tube, while Figure 15 shows the variation of temperature distribution in the 45# steel mold and CFRP pipe. In the initial heating stage, the region of the pipe corresponding to the coil experiences a temperature rise first. Once the steady state is reached, the temperature distribution in the CFRP circular tube exhibits a rectangular pattern, with the majority of the temperature concentrated at the center of the coil. By comparing with Figure 11 and Figure 12, it can be observed that the temperature distribution patterns obtained in the experiments are consistent with the simulation results. Although there is a slight discrepancy in temperature values due to the limited resolution of the infrared thermal camera, it falls within an acceptable range. Therefore, it can be concluded that the simulation in this study is accurate.

## 4. Discussion

**Figure 16:**
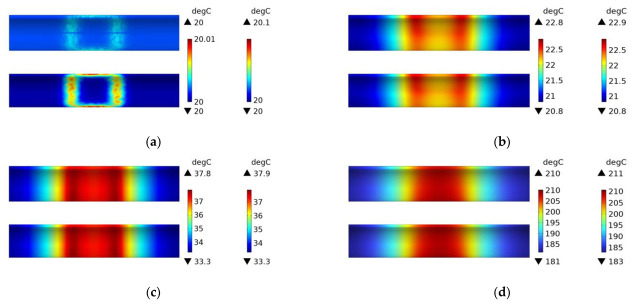
Temperature field distribution of 45# steel mold and winding structure (winding angle 89 degrees) CFRP circular tube; (**a**) 1 s, (**b**) 60 s, (**c**) 600 s, (**d**) steady state.

In this paper, electromagnetic induction heating simulations were conducted on three different structures (woven plain weave structure, fiber winding structure with winding angles of 89 degrees and 45 degrees) using both a 45# steel mold and an FRP mold. Figure 16 shows the temperature field distribution of CFRP circular tubes (wrapped at a winding angle of 89 degrees) on a 45# steel mold during the process of rotating induction heating, from initial heating to steady state. The temperature field distribution gradually transitions from a rectangular shape to high temperature on both sides and low temperature in the middle, and eventually reaches a steady state where the temperature is highest in the middle. The temperature field distribution is almost identical to Figure 12, which indicates that the temperature field distribution only depends on the material parameters of the metal mold when heating is applied to the metal mold, regardless of the CFRP structure. This is because the metal mold has much higher magnetic permeability than CFRP.

**Figure 17:**
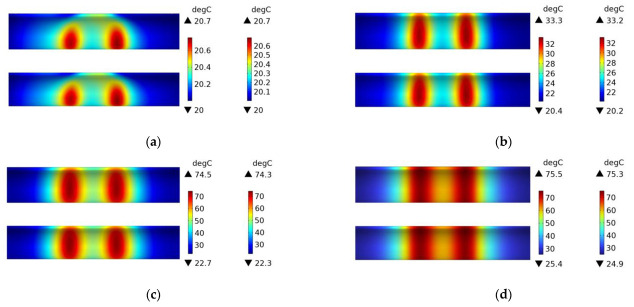
Temperature field distribution of FRP mold and winding structure (winding angle 89 degrees) CFRP circular tube; (**a**) 1 s, (**b**) 60 s, (**c**) 600 s, (**d**) steady state.

It can be seen from Figure 17 that the temperature field distribution is mainly concentrated on both sides, and the temperature difference between the middle and both sides is nearly 20 degrees Celsius at a steady state. This is because the CFRP tube with a winding angle of 89 degrees has few interwoven fibers in its structure, and most of them are almost parallel to each other. In the heating process, the temperature field is mainly concentrated in the area directly facing the two sides of the coil. Since the CFRP tube with a winding angle of 89 degrees has a small thermal conductivity in the axial direction, the middle temperature has not increased significantly at a steady state. Compared with Figure 11, the temperature field distribution at the steady state in Figure 17 is very uneven, and the maximum temperature value is not much different from that at 600 s, indicating that structures with fewer intersection points can heat up to a steady state more quickly.

**Figure 18:**
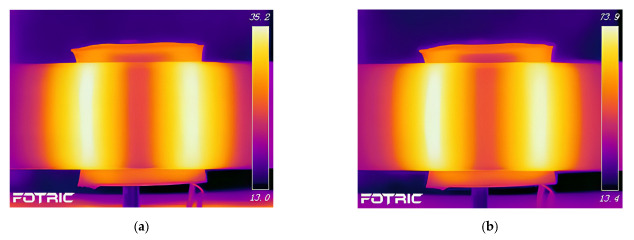
Experimental temperature field distribution of FRP mold and winding structure (winding angle 89 degrees) CFRP circular tube; (**a**) 60 s, (**b**) 600 s.

Figure 18 shows the experimental results of heating the FRP mold and winding structure (winding angle of 89 degrees) CFRP circular tube, which demonstrates the accuracy of the simulation compared to Figure 17. The temperature field distribution at 60 s and 600 s is displayed in the figure.

**Figure 19:**
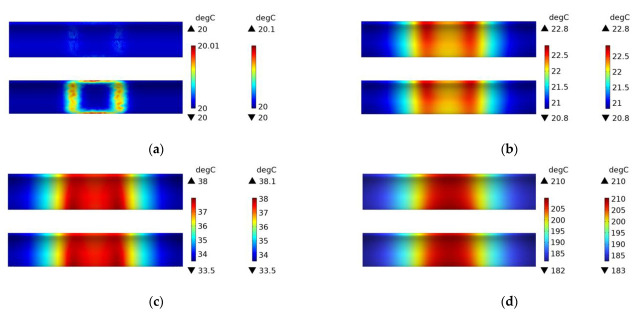
Temperature field distribution of 45# steel mold and winding structure (winding angle 45 degrees) CFRP circular tube; (**a**) 1 s, (**b**) 60 s, (**c**) 600 s, (**d**) steady state.

Figure 19 shows the temperature field distribution of the CFRP circular tube (winding angle of 45 degrees) with a 45# steel mold and winding structure during the induction heating process from initial heating to steady state. Compared with Figure 12 and Figure 16, it can be observed that the temperature field distribution in these three figures is almost identical, which once again demonstrates that the metal mold is the primary source of heat generation during electromagnetic induction heating.

**Figure 20:**
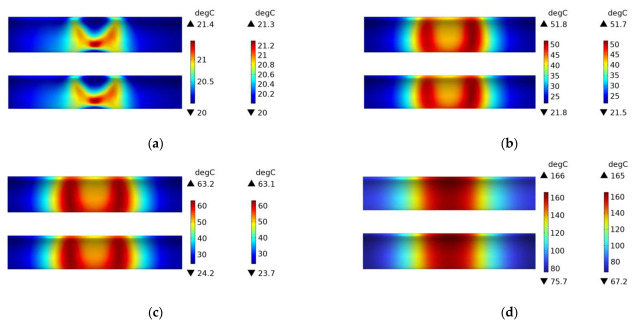
Temperature field distribution of FRP mold and winding structure (winding angle 45 degrees) CFRP circular tube; (**a**) 1 s, (**b**) 60 s, (**c**) 600 s, (**d**) steady state.

Figure 20 shows the temperature field distribution of the CFRP circular tube (winding angle of 45 degrees) with an FRP mold and winding structure during the induction heating process from initial heating to steady state. At 1 s, it can be clearly seen from the figure that the temperature is mainly distributed in the area opposite to the centerline of the coil, and it presents an “X” cross shape, with the highest temperature at the intersection point, which is consistent with the characteristics of the 45-degree winding angle. From 60 s onwards, the temperature field gradually shifts from the center to both sides, and with increasing time, the temperature values in the corresponding areas on both sides continue to rise, and the temperature field distribution range also expands along the axis of the circular tube, until steady state is reached. At a steady state, the temperature at the center is the highest and relatively uniform, and the temperature field spreads towards both sides. Compared with Figure 11 and Figure 17, it can be found that when the CFRP circular tube is used as the heat source, the plain weave structure with more fiber interweaving points and the winding structure with a winding angle of 45 degrees have very similar temperature fields at the final steady state, and the temperature field distribution at the center is relatively uniform. However, the steady-state temperature field of the winding structure with a winding angle of 89 degrees is not very uniform, which indicates that the structure of CFRP has a significant influence on the temperature field distribution. Compared with Figure 11, Figure 12, Figure 16, Figure 17 and Figure 19, regardless of the material of the mold and the structure of the CFRP circular tube, when heated under a specific arc-shaped coil, the temperature field distribution is basically related to the two vertical edges of the coil, and before reaching steady state, the temperature values of the two vertical edges are relatively high. It can be found that the coil shape has a crucial influence on the temperature field distribution, which has certain predictive significance for the temperature field distribution of heated CFRP components.

**Figure 21:**
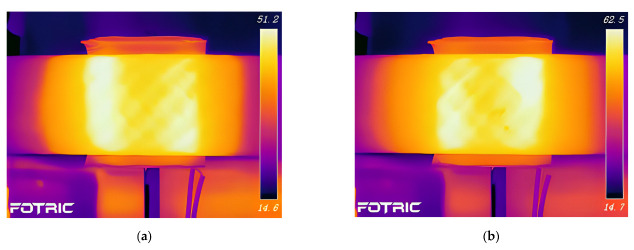
Experimental temperature field distribution of FRP mold and winding structure (winding angle 45 degrees) CFRP circular tube; (**a**) 60 s, (**b**) 600 s.

Figure 21 shows the experimental results of the induction heating of a CFRP tube wrapped around an FRP mold with a winding angle of 45 degrees. Comparing it with the simulation in Figure 20, it can be observed that the distribution and values of the temperature field are consistent between the two, which confirms the accuracy of the simulation.

## 5. Conclusions

A finite element analysis model was developed based on the electromagnetic-thermal coupling characteristics of carbon fiber reinforced polymer (CFRP) for the induction heating of a rotating CFRP circular tube. The model simulated and calculated the magnetic flux density, eddy current loss, and temperature field distribution of the plain weave CFRP circular tube on both the moldless and FRP molds, explaining the heating law of the CFRP circular tube in the electromagnetic induction heating process. The model analyzed the effects of the presence or absence of molds on the magnetic field, eddy current, and temperature field, and compared the results. It was found that when heated on the FRP mold, the heat source was concentrated on the CFRP, and therefore the distribution and numerical values of eddy current loss and temperature field were basically consistent with those without molds. The temperature field distribution of the FRP mold and plain weave CFRP circular pipe under the influence of the magnetic field was experimentally characterized, and the accuracy of the established finite element model was verified.

Based on the coupling relationship between the magnetic field and temperature field distribution established in this paper, electromagnetic induction heating simulations of 45# steel molds and plain weave CFRP circular tubes were carried out. The effects of different molds on the magnetic field, eddy current loss, and temperature field distribution of plain weave CFRP circular tubes were compared. It was found that the heat source was mainly concentrated on the metal mold, and the temperature field distribution of 45# steel molds and CFRP circular tube was more uniform than that of the FRP mold, which was due to the much greater relative permeability of the metal mold compared to CFRP and FRP. The paper also analyzed the effects of different molds on the heating law of CFRP circular tube with a fiber winding structure (winding angle of 89 degrees), and demonstrated that the uneven temperature field distribution of this structure when heated on the FRP mold was not directly related to the number of crossing points, and the accuracy of the model was verified through experiments.

A comparative analysis was conducted on the influence of different molds on the temperature field distribution of a 45-degree fiber-wound CFRP circular tube structure. In comparison with the other two structures, it was found that the temperature field distribution of the CFRP circular tube wound with an FRP mold and a winding angle of 45 degrees was significantly different. The temperature first increased at the center of the coil and exhibited an “X”-shaped cross, which is in line with the characteristics of a 45-degree winding angle. Subsequently, the temperature field gradually shifted to the two parallel vertical sides corresponding to the coil and diffused through the two vertical sides until reaching a steady state. Moreover, it was found that the shape of the coil had a significant influence on the temperature field distribution in comparison with different structures and molds. The accuracy of the model was verified through experiments. This paper discusses the feasibility of combining electromagnetic induction heating technology with in-situ curing molding, providing theoretical guidance for practical industrial production.

## Figures and Tables

**Table 1:** Material parameter table.

	CFRP Circular Tubes			45# Steel Mold	FRP Mold	Copper Coil	Air
	Plain weave	89 degrees	45 degrees	N/A	N/A	N/A	N/A
Relative permeability	1			200	1	1	1
Relative dielectric constant	6.8			1	4.2	1	1
Density(kg/m^3^)	1500			7870	2000	8960	N/A
Constant pressure heat capacity (J/(kg·K))	1000			486	923.8	385	N/A
Conductivity (S/m)	30, 1800, 1800	30, 1800, 30	30, 1800, 1800	1.62 × 10^6^	0	6 × 10^7^	1
Thermal conductivity (W/(m·K))	2, 8, 8	2, 8, 2	2, 8, 8	49.8	0.287	400	N/A

## Data Availability

Not applicable.
